# The Impact of KRAS Status on Long‐Term Outcomes After Thermal Ablation and Hepatic Resection for Liver‐Only Colorectal Metastases: A New Clue for Tailoring Surgical Strategy on Tumor Biology?

**DOI:** 10.1002/wjs.12616

**Published:** 2025-05-08

**Authors:** Fabio Giannone, Federico Sangiuolo, Gianluca Cassese, Marco Palucci, Celeste Del Basso, Alfonso Lapergola, Giorgio Badessi, Reza Kianmanesh, Patrick Pessaux, Rami Rhaiem, Fabrizio Panaro

**Affiliations:** ^1^ Robotic, Oncologic and HPB Surgery Azienda Ospedaliero‐Universitaria SS. Antonio e Biagio e Cesare Arrigo Alessandria Italy; ^2^ Department of Visceral and Digestive Surgery University Hospital of Strasbourg Strasbourg France; ^3^ Department of Research and Innovation (DAIRI) Azienda Ospedaliero‐Universitaria SS. Antonio e Biagio e Cesare Arrigo Alessandria Italy; ^4^ Department of Health Science University of Eastern Piedmont Alessandria Italy; ^5^ Department of HBP and Digestive Oncological Surgery Robert Debré University Hospital University Reims Champagne‐Ardenne Reims France; ^6^ Institut de Recherche sur les Maladies Virales et Hépatiques U1110 Université de Strasbourg Strasbourg France

**Keywords:** colorectal liver metastases, liver resection, outcomes, thermal ablation

## Abstract

**Introduction:**

KRAS mutation is a negative prognostic factor for colorectal liver metastases (CRLM). Thermal ablation (TA) is considered a valid alternative to liver resection (LR) for CRLM in selected cases. This study aims to investigate the influence of KRAS status on long‐term outcomes of TA during LR.

**Materials and Methods:**

This is a retrospective analysis of patients undergoing surgery for CRLM in two hepatobiliary centers. Patients were divided into two groups: LR or LR + TA, and long‐term results were investigated according to KRAS status.

**Results:**

220 patients were included, of whom 74 (33.6%) were KRAS mutated. TA was performed in association with LR in 42 mutated (*mKRAS*) tumors (56.7%). Multivariate analysis in *mKRAS* patients showed that synchronous disease (*p* = 0.014), performing TA (*p* = 0.044), performing two or more TA (*p* = 0.032), and N2 status (*p* < 0.001) were independently associated with DFS. TA was more frequently associated with a liver‐only recurrence, both in mutated and *wt* tumors, but with a higher risk in *mKRAS* (*p = 0.013* vs. *p = 0.048*).

**Conclusion:**

CRLM surgical treatment should be tailored to KRAS status because TA may potentially be less effective in mutated patients during surgical resection. This is even more important in the case of multiple ablations.

## Graphical abstract

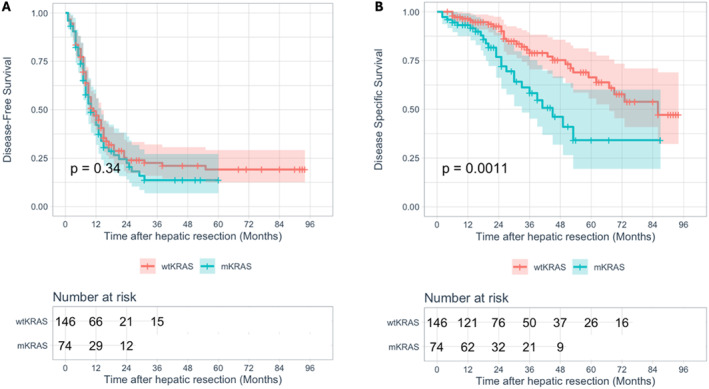

## 1 Introduction

Colorectal cancer (CRC) represents the second most common cancer worldwide, being a leading cause of cancer‐related mortality worldwide [1]. The main driver of tumor prognosis is represented by distant metastasis, with the liver accounting for the most commonly involved site. Indeed, colorectal liver metastases (CRLM) occur in more than 50% of the patients with CRLM. Surgical resection is the cornerstone treatment for CRLM with no extrahepatic disease, always within multimodal management together with systemic chemotherapy (CT). In patients undergoing liver surgery for CRLM, the 5‐year overall survival (OS) rate has been reported to be as high as 50% [2, 3]. According to most current Western guidelines, thermal ablation (TA) is a valid alternative option for CRLM nodules smaller than 3 cm [4, 5]. A recent randomized controlled trial has shown that TA is associated with similar survival outcomes, lower postprocedural complications, and a shorter length of stay when compared to surgery [6]. Furthermore, TA has been reported to be effective also when associated with surgical resection itself in order to perform an effective parenchymal‐sparing treatment in both open and laparoscopic approaches [7, 8]. Nonetheless, local recurrence has always been shown to be the most important issue when performing TA, with many prognostic risk factors that have been proposed, such as type of ablation, completeness of the ablation, and tumor burden. However, this argument is still a matter of debate [9, 10]. Recent studies have revealed that somatic mutations in genes such as *KRAS* and BRAF are associated with poor clinical outcomes in patients with CRLM [11, 12, 13, 14, 15]. Mutations in KRAS are found in up to 30% of the patients with CRLM [16]. However, few studies have investigated the association between the mutational status of *KRAS* and the results of TA associated with liver resection (LR).

This study aims to investigate the possible influence of *KRAS* mutation on the oncologic outcomes of TA associated with LR.

## 2 Methods

### 2.1 Study Design

This is a retrospective analysis based on prospectively maintained databases of two high‐volume hepatobiliary French centers [17]. This study was aligned with the ethical standards of the Declaration of Helsinki [18] and no specific informed consent was obtained given the retrospective and observational nature of the analysis. Principles of Strengthening the Reporting of Observational Studies in Epidemiology (STROBE) guidelines were followed for data curation and analysis [19]. No funding source was used for this study. All consecutive patients undergoing surgery between January 1, 2015, and December 31, 2022, for nonrecurrent CRLM were initially included in the analysis. Exclusion criteria were as follows: (i) disease spread to other organs other than the liver before LR (M1*b* according to the TNM eighth edition [20]), (ii) peritoneal dissemination (M1*c*), (iii) unknown *KRAS* status, and (iv) incomplete data or follow‐up (FU) inferior to 12 months. In‐hospital deaths were also excluded from the analysis.

After the first screening, patients with a planned two‐stage hepatectomy who did not undergo the second operation for disease progression or could not benefit from colic resection for the same reason in a liver‐first strategy were not included in the statistical analysis. Final cases included both patients undergoing LR or combined resection and TA (LR + TA group). Patients with a two‐stage hepatectomy who benefitted from at least one ablation during one of the stages were included in the LR + TA group. Genetic assessment in resected liver specimens always considered *KRAS*, *NRAS*, and *BRAF* status, performed through next‐generation sequencing. *KRAS* status was assessed to study patients with or without this type of mutation. In these two cohorts, patients were compared in terms of baseline characteristics and long‐term outcomes according to TA performance.

### 2.2 TA Indications and Technique

Indications for TA during hepatic resection in our centers were recently described [8]. Indications were always validated at the oncological multidisciplinary meeting. Ablations were performed by an experienced surgeon with experience in more than 50 TA procedures. The radiofrequency or microwave ablation system was used depending on vascular proximity and device availability. Electrode and active tip lengths were chosen according to tumor location and size, and US was performed to guide tip placement.

### 2.3 Perioperative Variables

All clinicopathological features were collected and compared between the two cohorts. Nodule number and maximum size were estimated from lesions found at histological assessments and any putative metastasis submitted to TA treatment. Tumor number was classified into two groups: fewer than 4 or ≥ 4 lesions, this being a cut‐off prognostic factor for recurrence. These variables, as well as margin status, were evaluated considering both stages in two‐stage hepatectomies. Similarly, perioperative and postoperative features considered both surgical resections, except for type of approach and extension of hepatectomy, which were exclusively related to the second stage. Postoperative complications were classified according to the Clavien–Dindo classification and included 90‐day morbidity and mortality [21]. Resection margin was considered positive if tumor cells were present at ≤ 1 mm. Follow‐up included a CT scan with CEA and CA 19‐9 measurement every 3 months for the first 2 years and then every 6 months up to 5 years. Data regarding the first site of recurrence were collected in all patients and classified into liver‐only recurrence or extrahepatic/multimetastatic relapse. This factor was then matched with the two groups and with other main variables to assess a possible influence on liver recurrence.

### 2.4 Statistical Analysis

Continuous data are displayed as median with standard deviation (SD) and compared using Student's t‐test or Mann–Whitney *U* test if the distribution was normal. Categorical data are reported with relative proportions (%), with distribution between groups assessed using the χ2 test and Yates' correction if necessary. Kaplan–Meier curves were constructed, and survival outcomes were compared using a log‐rank test for categorical variables and a Cox test for continuous data. Hazard ratio (HR) and its 95% confidence interval (CI) were reported. Variables with a *p*‐value equal to or lower than 0.05 in the univariate analysis were included in the final multivariate model. Finally, a multinomial logistic regression was performed to assess the association between specific site of recurrence and the most important variables, including performing TA, according to KRAS status. All tests were 2‐tailed, and the level of significance was set at *p* < 0.05. All statistical computations were performed using SPSS (SPSS Statistics for Macintosh, version 26.0, IBM Corp) and R (R project for statistical computing, version 4.2.2 for Mac, R Core Team).

## 3 Results

### 3.1 General Features

A total of 231 patients from the common database met the inclusion criteria set for this study. The STROBE flowchart is shown in Supporting Figure 1. Of these, 11 (4.8%) patients experienced disease progression before undergoing colic resection or second stage and were thus excluded from the analysis. These patients were mostly *KRAS* mutated (*mKRAS*, *n* = 8, 72.7%) and in the LR + TA group (*n* = 10, 90.9%).

The final cohort consisted, therefore, of 220 patients, of whom 74 (33.6%) showed a *KRAS* mutation at genetic assessment. Main features compared according to the *KRAS* status are shown in Supporting Table 1. *BRAF* and *NRAS* mutations were less frequently encountered, with an incidence of 9 (4.1%) and 16 (7.3%) cases, respectively. TA was associated with LR in 73 out of the 146 (50%) wild‐type (*wt*) *KRAS* tumors and in 42 out of the 74 *mKRAS* patients (56.7%). Median number of TA performed was 2 (range: 0–9) in the *wtKRAS* group, with 29 patients (19.9%) benefitting from only one TA and 44 (30.1%) undergoing two or more ablations during liver resection. In *mKRAS* patients, median number of TA was 1 (range: 0–8), with 20 (27%) and 22 patients (29.7%) undergoing one or more ablations, respectively. Perioperative variables of the two groups (LR vs. LR + TA) compared according to their mutational status are shown in Table 1. In *mKRAS* patients, the two groups, LR and TA + LR, were similar in all the variables assessed, except for CLRM distribution (*p = 0.030*). On the contrary, in *wt* patients, the TA + LR group showed a more aggressive disease compared to those undergoing hepatic resection alone. CRLM in which at least 1 TA was performed were, in fact, more often synchronous (*p = 0.004*), with a higher preoperative CEA (*p = 0.047*), with more than 4 nodules (*p < 0.001*), with a bilobar distribution (*p < 0.001*), and with a higher T stage of the primary tumor (*p = 0.021*).

**TABLE 1:** Clinicopathological features of the two groups (LR and LR + TA) according to KRAS mutational status.

Variables	*Wild‐type KRAS, n = 146*			*Mutated KRAS, n = 74*		
	Liver resection *n = 73*	Thermal ablation + liver resection *n = 73*	*p*	*Liver resection n = 32*	Thermal ablation + liver resection *n = 42*	*p*
	*n (%)*			*n (%)*		
Median age, yrs (IQR)	63 (56–71)	66 (54.5–73)	0.528	67 (61–73.7)	67 (55–71)	0.709
Sex						
Male	48 (65.8)	50 (68.5)	0.860	20	28	0.900
Female	25 (34.2)	23 (31.5)		12	14	
Median BMI, kg/m^2^ (IQR)	25.4 (22.2–28)	24.9 (22.9–27.7)	0.935	24.5 (21.8–27)	25 (22.9–29.4)	0.244
ASA						
I	5 (6.8)	4 (5.5)	0.788	2	2	0.364
II	36 (49.3)	33 (45.2)		20	20	
III	32 (43.8)	36 (49.3)		10	20	
Status at diagnosis						
Synchronous	37 (50.7)	55 (75.3)	***0.004***	18	30	0.267
Metachronous	36 (49.3)	18 (24.7)		14	12	
Site of primary tumor						
Right colon	13 (17.8)	16 (21.9)	0.495	11	17	0.559
Transverse colon	2 (2.7)	5 (6.8)		—	2	
Left colon	35 (47.9)	28 (38.4)		12	13	
Rectum	23 (31.5)	24 (32.9)		9	10	
Primary tumor resection						
Prior to LR	60 (82.2)	57 (78)	0.680	26	32	0.060
Combined with LR	5 (6.8)	8 (11)		2	9	
Liver‐first strategy	8 (11)	8 (11)		4	1	
Preoperative chemotherapy						
No	16 (21.9)	11 (15.1)	0.394	4	3	0.705
Yes	57 (78.1)	62 (84.9)		28	39	
CEA (median), ng/mL (IQR)	2.9 (1.8–4.6)	5 (2–29.4)	***0.047***	7.5 (3–178)	8 (3.7–46.5)	0.886
Number of nodules						
< 4	51 (69.9)	24 (32.9)	***< 0.001***	21	19	0.132
≥ 4	22 (30.1)	49 (67.1)		11	23	
Distribution						
Unilobar	45 (61.6)	24 (32.9)	***< 0.001***	18	12	***0.030***
Bilobar	28 (38.4)	49 (67.1)		14	30	
Largest nodule (mm), median (IQR)	35 (20–60)	25 (15.5–40)	***0.004***	28 (22.2–40)	32 (20–47.5)	0.510
Type of hepatectomy						
Minor	40 (54.8)	44 (60.3)	0.615	22	24	0.437
Major	33 (45.2)	29 (39.7)		10	18	
Approach						
Open	51 (69.9)	53 (72.6)	1	22	36	0.200
MI	22 (30.1)	20 (27.4)		10	6	
*R* status						
R0	66 (90.4)	62 (84.9)	0.450	27	36	1
R1	7 (9.6)	11 (15.1)		5	6	
Primary *T* status						
Complete response	3 (4.1)	1 (1.4)	***0.021***	—	2	0.521
1	3 (4.1)	4 (5.5)		—	—	
2	14 (19.2)	2 (2.7)		4	3	
3	41 (56.2)	51 (69.9)		21	26	
4	12 (16.4)	15 (20.5)		7	11	
Primary *N* status						
0	29 (39.7)	27 (37)	0.943	13	10	0.081
1	25 (34.2)	26 (35.6)		14	16	
2	19 (26)	20 (27.4)		5	16	

*Note:* The bold‐italic values indicate statistically significant.

Abbreviations: ASA, American Society of Anesthesiologists; BMI, body mass index; CEA, carcinoembryonic antigen; IQR, interquartile range; KRAS, Kirsten rat sarcoma virus; LR, liver resection; MI, minimally invasive; TA, thermal ablation.

### 3.2 Analysis of Long‐Term Outcomes

**FIGURE 1:**
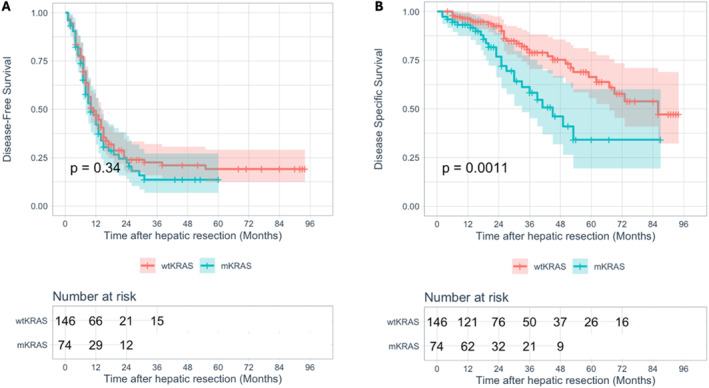
Kaplan–Meier curves of DFS (A) and DSS (B) comparing patients with or without a KRAS mutation.

**FIGURE 2:**
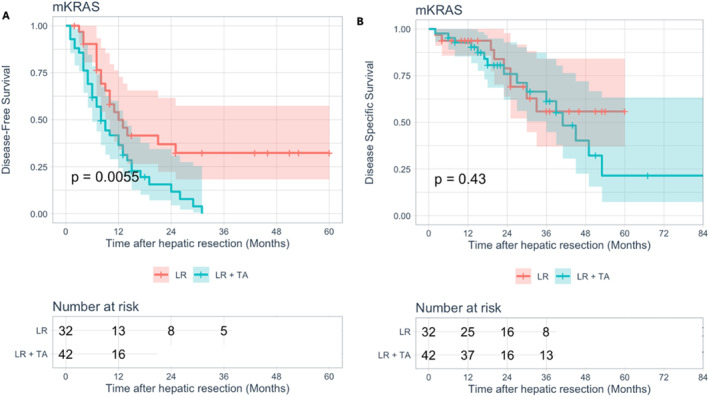
Kaplan–Meier curves of DFS (A) and DSS (B) comparing the two groups (liver resection alone vs. liver resection + thermal ablation) in *mKRAS* patients. LR: liver resection, TA: thermal ablation.

Overall median disease‐free survival (DFS) and disease‐specific survival (DSS) were 10 months (95% CI: 2–59.7) and 23 months (95% CI: 6–83.6), respectively. Tumor‐related death occurred in 55 patients (25%), whereas 158 (71.8%) experienced disease recurrence. No difference in DFS was observed between mutated and not mutated patients (median DFS: 10 months, 95% CI: 7–13 vs. 11 months, 95% CI: 8.2–13.8, *p = 0.337*. Figure 1A), whereas *mKRAS* tumors showed a worse DSS (median DSS: 45 months, 95% CI: 32.1–57.8 vs. 86 months, 95% CI not reached, *p = 0.001*. Figure 1B). Comparison between LR alone and TA + LR according to *KRAS* status was then performed. In *wt* patients, combining TA and resection was associated with an increased risk of recurrence (*p < 0.001*, Supporting Figure 2A) but with similar survival rates (*p = 0.598*, Supporting Figure 2B). In the presence of *mKRAS*, patients undergoing TA during LR showed worse DFS (median DFS: 8 months, 95% CI: 5.6–10.4 vs. 12 months, 95% CI: 7.5–16.5, *p = 0.006*. Figure 2A) but similar DSS (median DFS: 41 months, 95% CI: 32.1–49.9 vs. not reached, *p = 0.431*. Figure 2B). Univariate and multivariate analysis for DFS and DSS in *mKRAS* and *wtKRAS* patients were then performed (Tables 2 and 3). Two different multivariate models were constructed to avoid data collinearity between “TA performance” and “number of TA.” In mutated tumors (Table 2), factors independently associated with the risk of recurrence were synchronous disease (metachronous vs. synchronous, HR: 0.46, 95% CI: 0.25–0.85; *p = 0.014*), TA performance (HR: 1.83, 95% CI: 1.02–3.28; *p = 0.044*), performing two or more TA (HR: 2.11, 95% CI: 1.06–4.18, *p = 0.032*), and N2 status in the primary tumor (HR: 4.15, 95% CI: 1.86–9.24, *p < 0.001*). For DSS, only N2 status (HR: 2.66, 95% CI: 0.98–7.18; *p = 0.045*) and positive resection margin (HR: 2.74, 95% CI: 1.05–7.12; *p = 0.039*) were found to be independent predictors. In *wt* diseases (Table 3), combining LR and TA was not an independent predictor of recurrence (HR: 1.45, 95% CI: 0.95–2.22; *p = 0.086*) or tumor‐related death (*p = 0.600* at univariate analysis, not shown), whereas positive resection margin (HR: 2.61, 95% CI: 1.57–4.34; *p < 0.001*) and number of nodules (HR: 1.7, 95% CI: 1.11–2.58; *p = 0.014*) were independently associated with a higher risk of recurrence.

**TABLE 2:** Univariate and multivariate Cox regression analysis of prognostic factors for disease‐free survival and disease‐specific survival in *mKRAS* patients.

		*Disease‐free survival*		*Disease‐specific survival*	
Variable	Category	Hazard ratio (95% CI)	*p*	Hazard ratio (95% CI)	*p*
*Univariate analysis*					
Age	Continuous data	0.98 (0.95–1)	0.087	0.96 (0.93–1)	0.057
Sex	Female versus male	0.83 (0.47–1.46)	0.526	1.12 (0.49–2.55)	0.786
BMI	Continuous data	1 (0.95–1.06)	0.910	1 (0.92–1.1)	0.920
ASA	II versus I	0.86 (0.26–2.86)	0.837	0.94 (0.36–2.5)	0.465
	III versus I	1.02 (0.3–3.4)		1.35 (0.73–2.52)	
Status at diagnosis	Metachronous versus synchronous	0.37 (0.2–0.67)	***0.001***	0.41 (0.17–0.98)	***0.046***
Site of primary tumor	Transverse versus right colon	1.71 (0.23–12.9)	0.941	5.05 (0.58–43.9)	0.243
	Left versus right colon	0.96 (0.51–1.8)		0.56 (0.21–1.53)	
	Rectum versus right colon	1.08 (0.56–2.1)			
				0.73 (0.27–1.94)	
Previous primary tumor resection	Yes versus no	1.32 (0.64–2.71)	0.453	0.73 (0.25–2.13)	0.561
Preoperative chemotherapy	Yes versus no	2.8 (0.86–9.09)	0.087	1.29 (0.38–4.36)	0.681
Number of nodules	≥ 4 versus < 4	1.85 (1.08–3.17)	***0.025***	2.08 (0.93–4.63)	0.074
Distribution	Bilobar versus unilobar	1.97 (1.11–3.5)	***0.020***	1.45 (0.65–3.27)	0.365
Tumor size (largest, mm)	Continuous data	1 (0.99–1.02)	0.581	0.99 (0.96–1.02)	0.550
Preoperative CEA (ng/mL)	Continuous data	1 (0.99–1)	0.479	1 (0.99–1.01)	0.177
TA performed	Yes versus no	2.16 (1.22–3.84)	***0.008***	1.39 (0.61‐3‐14)	0.435
Number of TA	1 versus 0	1.91 (0.99–3.69)	***0.021***	1.25 (0.48–3.25)	0.664
	≥ 2 versus 0	2.52 (1.29–4.94)		1.55 (0.6–4.04)	
Type of TA	MWA versus RFA	1.05 (0.79–1.4))	0.727	1.1 (0.72–1.69)	0.651
Type of hepatectomy	Major versus minor	1.61 (0.93–2.79)	0.091	1.6 (0.72–3.54)	0.244
Approach	MI versus open	0.53 (0.26–1.05)	0.070	1.46 (0.65–3.28)	0.354
Severe postoperative complications (CD ≥ 2)	Yes versus no	1.12 (0.7–1.77)	0.639	0.78 (0.42–1.44)	0.426
*R* status	R1 versus R0	1.43 (0.67–3.04)	0.351	2.94 (1.15–7.53)	***0.025***
ypT (TNM, 8th ed.)	3 versus others	3.36 (1.24–9.12)	0.057	2.67 (0.77–9.28)	0.256
	4 versus others	2.64 (0.93–7.49)		1.7 (0.37–7.86)	
ypN (TNM, 8th ed.)	1 versus 0	1.35 (0.67–2.73)	***< 0.001***	0.84 (0.29–2.41)	***0.033***
	2 versus 0	5.41 (2.48–11.8)		2.78 (1.03–7.47)	
*Multivariate analysis*					
Status at diagnosis	Metachronous versus synchronous	0.46 (0.25–0.85)	***0.014***	0.71 (0.25–2)	0.519
Number of nodules	≥ 4 versus < 4	1.11 (0.62–1.97)	0.733	—	—
Distribution	Bilobar versus unilobar	0.93 (0.46–1.88)	0.832	—	—
TA performed^a^	Yes versus no	1.83 (1.02–3.28)	***0.044***	—	—
Number of TA^a^	1 versus 0	1.68 (0.86–3.3)	0.130	—	—
	≥ 2 versus 0	2.11 (1.06–4.18)	***0.032***		
*R* status	R1 versus R0	—	—	2.74 (1.05–7.12)	***0.039***
ypT (TNM, 8th ed.)	3 versus others	2.31 (0.79–6.74)	0.125	—	—
	4 versus others	1.36 (0.43–4.32)	0.596		
ypN (TNM, 8th ed.)	1 versus 0	1.25 (0.61–2.55)	0.535	0.84 (0.29–2.39)	0.740
	2 versus 0	4.15 (1.86–9.24)	***< 0.001***	2.66 (0.98–7.18)	***0.045***

*Note:* The bold‐italic values indicate statistically significant.

Abbreviations: ASA, American Society of Anesthesiologists; BMI, body mass index; CEA, carcinoembryonic antigen; CD, Clavien–Dindo; CI, confidence interval; KRAS, Kirsten rat sarcoma virus; MI, minimally invasive; MWA, microwave ablation; RFA, radiofrequency ablation; TA, thermal ablation.

^a^ Two different models were constructed to avoid collinearity between the performance of TA and the number of TA.

**TABLE 3:** Multivariate Cox regression analysis of prognostic factors for disease‐free survival in *wtKRAS* patients.

Variable	Category	Hazard ratio (95% CI)	*p*
*Multivariate analysis*			
Status at diagnosis	Metachronous versus synchronous	0.85 (0.55–1.34)	0.492
Preoperative chemotherapy	Yes versus no	1.44 (0.9–2.6)	0.224
Number of nodules	≥ 4 versus < 4	1.7 (1.11–2.58)	***0.014***
Distribution	Bilobar versus unilobar	1.05 (0.62–1.8)	0.846
TA performed	Yes versus no	1.45 (0.95–2.22)	0.086
Approach	MI versus open	0.91 (0.52–1.58)	0.741
*R* status	R1 versus R0	2.61 (1.57–4.34)	***< 0.001***

*Note:* The bold‐italic values indicate statistically significant.

Abbreviations: CI, confidence interval; KRAS, Kirsten rat sarcoma virus; MI, minimally invasive; TA, thermal ablation.

### 3.3 Analysis of Risk of Liver‐Only Recurrence

Of the whole cohort, 103 of *wt* patients (70.5%) and 55 of the mutated patients (74.3%) experienced disease recurrence. Liver‐only disease relapse was more frequent in tumors not harboring a KRAS mutation (38.4% vs. 28.4% in *mKRAS*). When performing a multinomial logistic regression to identify risk factors of liver‐only recurrence compared to systemic/multisite relapse, no specific predictors were found, independently from *KRAS* status (Table 4). On the contrary, performing a TA was more frequently associated with a hepatic recurrence compared to nonrecurrent diseases, both in mutated and *wt* patients, but with a higher risk in the first group (HR: 0.12, 95% CI: 0.24–0.64; *p = 0.013* vs. HR: 0.4, 95% CI: 0.16–0.99; *p = 0.048*).

**TABLE 4:** Multinomial logistic regression of variables associated with the risk of liver‐only recurrence in *mKRAS* and *wtKRAS* patients.

		*Mutated KRAS*		*Wild‐type KRAS*	
Variable	Category	Hazard ratio (95% CI)	*p*	Hazard ratio (95% CI)	*p*
*Systemic* versus *liver‐only recurrence*					
Status at diagnosis	Metachronous versus synchronous	1.29 (0.35–4.76)	0.702	1.68 (0.66–4.27)	0.272
Number of nodules	≥ 4 versus < 4	0.62 (0.15–2.55)	0.511	1.06 (0.39–2.88)	0.906
Distribution	Bilobar versus unilobar	2.28 (0.5–10.44)	0.287	1.12 (0.43–2.93)	0.818
TA performed	Yes versus no	0.31 (0.08–1.23)	0.095	0.52 (0.22–1.24)	0.141
*R* status	R1 versus R0	7.4 (0.72–75.6)	0.091	—	—
ypN (TNM, 8th ed.)	Overall status	0.58 (0.25–1.35)	0.209	1 (0.61–1.64)	0.998
*No recurrence* versus *liver‐only recurrence*					
Status at diagnosis	Metachronous versus synchronous	1.53 (0.31–7.72)	0.604	0.93 (0.36–2.45)	0.888
Number of nodules	≥ 4 versus < 4	0.7 (0.11–4.69)	0.716	0.65 (0.23–1.85)	0.423
Distribution	Bilobar versus unilobar	0.77 (0.12–5.02)	0.781	0.5 (0.18–1.36)	0.176
TA performed	Yes versus no	0.12 (0.24–0.64)	***0.013***	0.4 (0.16–0.99)	***0.048***
*R* status	R1 versus R0	9.3 (0.52–164.7)	0.130	—	—
ypN (TNM, 8th ed.)	Overall status	0.2 (0.06–0.65)	***0.007***	0.84 (0.5–1.43)	0.532

*Note:* The bold‐italic values indicate statistically significant.

Abbreviations: CI, confidence interval; KRAS, Kirsten rat sarcoma virus; TA, thermal ablation.

## 4 Discussion

Parenchymal‐sparing liver resection is a valid option to major resection for treating CRLM, especially when patient conditions or FLR represent a potential issue [22, 23, 24]. The oncologic adequacy of this approach is mainly connected to the steady and progressive improvements in the chemotherapeutic field over the years, allowing the expansion of the boundaries in the surgical indications. Undoing the maximum limit of resectable liver metastasis and reducing the surgical safety margin up to the introduction of the R1 vascular concept are some of the achievements gained in parallel with the introduction of more effective and targeted CT [25, 26]. In this setting, thermal ablation became a valid alternative for small centrohepatic lesions in order to reduce the sacrifice of liver parenchyma and surgical morbidity, while maintaining similar oncologic outcomes [6]. However, as the size and number of the treated lesions increase, the recurrence rate could worsen, mainly due to the risk of microscopic residual or insufficient ablation margin [8, 27]. This also happens in biologically hostile diseases, where the classic 1‐mm cut‐off should not be oncologically safe during surgical resection, which could be associated with tumor aggressiveness and a low response to chemotherapy [28, 29, 30]. Therefore, the more aggressive and poorly responsive to therapy the tumor is, the greater the risk of recurrence after thermal ablation. Among all the genetic mutations frequently explored in CRC, *KRAS* is surely one of the most important, being largely associated with poorer outcomes [31, 32]. Although TA effectiveness is widely demonstrated in the literature, the concept of TA associating oncologic outcomes and *KRAS* status is poorly explored in large cohorts [33, 34]. In a cohort of 92 patients, Odisio et al. demonstrated that *mKRAS* is associated with an earlier and higher rate of local tumor progression in patients undergoing ablation for CRLM [35]. Similarly, Calandri et al. reported that size > 2 cm, *mKRAS* status, and ablation margin < 10 mm were predictors of local recurrence after ablation for CRLM [36]. However, these studies focused on percutaneous thermal ablations and not on simultaneous surgical procedures. These two approaches differ significantly in clinical practice. For instance, the minimal ablation margin of 5 mm, described as necessary to avoid the increased risk of local progression after percutaneous TA, is not applicable during surgical resection [37]. Similarly, the Amsterdam group found the necessity of wider ablation margins in mutated *KRAS* patients treated with combined surgery and TA [34]. However, these authors also mixed percutaneous and surgical TA, with most of the patients treated through CT scan and with a post‐gesture imaging checking the results of the ablation. In this study, we confirmed that by comparing two groups with similar baseline prognostic factors, *mKRAS* status was associated with impaired recurrence rates when performing an US‐guided TA during LR compared to resection alone, and that this treatment was also an independent predictor of recurrence in multivariate analysis. In *wt* patients, this data was not confirmed, whereas a 1‐mm cut‐off resection margin was an independent prognostic factor. This means that US‐guided TA during liver resection is not always a safe procedure from an oncologic point of view, and one of the reasons is that the adequacy of the procedure and ablation margins cannot be checked with US due to thermal artifacts. This last report is not new in the literature and overlaps the previous concepts on TA. In fact, although *KRAS* mutation does not seem to be directly associated with the prevalence of positive margins [38], some authors have already reported that in surgical patients this mutation could influence the resection margin length needed, with the consequence that the widely adopted 1‐mm rule could not be sufficient in these aggressive tumors [39, 40]. We further assessed if tumors treated with TA had an increased risk of recurrence or, alternatively, if this depended on the number of ablations performed during the same procedure. It seems that this association is directly proportional, with a nonsignificant risk in the case of one ablation, while performing 2 TAs or more during LR impairs DFS rates, especially in mutated patients, with a risk 2.1 times higher of developing disease recurrence independently of other factors. This result is in contrast with what our group previously reported [8], but as we had already speculated, this could depend on tumor biology and surgeon indications.

Assessing the site of recurrence is another key point to better understand the relationship between ablation techniques and oncologic outcomes according to tumor biology. Previous studies have already reported how *KRAS* status was an essential variable in predicting local recurrence in percutaneous treatments [35, 36, 41]. In surgical scenarios, the correlation between liver‐only relapse and TA was confirmed by some studies only in cases of multiple ablations [42, 43], although our group did not find any association between the number of TA and liver‐only recurrence. How *KRAS* status influences this type of recurrence after TA during LR is poorly explored in the literature. In this study, TA was more frequently associated with liver‐only recurrence compared to those without disease relapse, both in mutated and in nonmutated *KRAS* tumors. This risk was, however, much higher in the first group, as an expression of a potential inadequacy of this treatment in a larger number of patients.

Some limitations have to be reported. Firstly, including two high‐volume hospitals allowed us to increase the sample size but reduced, at the same time, the homogeneity of the cohort. Indications were always validated at a multidisciplinary board, and strict criteria were applied, but techniques of TA and LR could be different between the two centers. Secondly, not all the different *KRAS* allele mutations have the same weight on patient prognosis [44], and this data was not always available in this analysis. Furthermore, this subanalysis would have made our conclusions even more fragmented due to the large number of variations. However, to strengthen our conclusions, data on other important mutations such as BRAF and NRAS were collected, but these had a low incidence in our cohort. Finally, the exact site of recurrence was always reported with the specific organ involved, but in the case of liver relapse, having the exact localization of the disease could have been a more truthful indicator of a noneffective treatment. In conclusion, patients harboring a KRAS mutation should be accurately selected before performing a parenchymal‐sparing surgical technique. TA indications must be extensively discussed in mutated patients because it is potentially less effective in terms of oncologic results and not always adequate when performed in a surgical scenario. This is even more important in the case of multiple ablations. Further prospective studies are needed to validate these findings.

## Author Contributions

**Fabio Giannone:** conceptualization, methodology, data curation, investigation, formal analysis, validation, visualization, writing – original draft, writing – review and editing, software. **Federico Sangiuolo:** data curation, methodology, formal analysis, writing – original draft, visualization, validation, software. **Gianluca Cassese:** methodology, formal analysis, software, visualization, validation, writing – review and editing. **Marco Palucci:** validation, visualization, writing – review and editing. **Celeste Del Basso:** validation, visualization, writing – review and editing. **Alfonso Lapergola:** validation, visualization, writing – review and editing, data curation. **Giorgio Badessi:** validation, visualization, writing – review and editing, data curation. **Reza Kianmanesh:** validation, visualization, writing – review and editing, supervision, methodology. **Patrick Pessaux:** validation, visualization, writing – review and editing, supervision, methodology. **Rami Rhaiem:** validation, visualization, writing – review and editing, data curation, methodology, writing – original draft, formal analysis, investigation. **Fabrizio Panaro:** validation, visualization, writing – review and editing, investigation, conceptualization, methodology, supervision.

## Conflicts of Interest

The authors declare no conflicts of interest.

## Declaration of AI and AI‐Assisted Technologies in the Writing Process

No specific AI tools/services were used for the preparation of this work.

## Supporting information

Supporting Information S1

## Data Availability

The data that support the findings of this study are available on request from the corresponding author. The data are not publicly available due to privacy or ethical restrictions.
