# Supplementary material for: The Impact of KRAS Status on Long‐Term Outcomes After Thermal Ablation and Hepatic Resection for Liver‐Only Colorectal Metastases: A New Clue for Tailoring Surgical Strategy on Tumor Biology?
Source: World J Surg. 2025 May 8;49(6):1573–82. doi: 10.1002/wjs.12616 (PMC12134197; doi:10.1002/wjs.12616)
Supplement: Supplementary file 1 — Supporting Information S1 [file WJS-49-1573-s001.docx]

**THE IMPACT OF KRAS STATUS ON LONG-TERM OUTCOMES AFTER THERMAL ABLATION AND HEPATIC RESECTION FOR LIVER-ONLY COLORECTAL METASTASES: A NEW CLUE FOR TAILORING SURGICAL STRATEGY ON TUMOR BIOLOGY?**

Fabio Giannone MD PhD^1,2,3^, Federico Sangiuolo MD^1^, Gianluca Cassese MD PhD ^1,3,4^, Marco Palucci MD^1,3^, Celeste Del Basso MD^1^, Alfonso Lapergola MD^2^, Giorgio Badessi MD^5^, Reza Kianmanesh MD^5^, Patrick Pessaux MD PhD ^2,6^, Rami Rhaiem MD PhD ^5^, Fabrizio Panaro MD PhD^1,3,4^

^1^ Robotic, Oncologic and HPB Surgery, Azienda Ospedaliero-Universitaria SS. Antonio e Biagio e Cesare Arrigo, Alessandria, Italy

^2^ Department of Visceral and Digestive Surgery, University Hospital of Strasbourg, Strasbourg, France

^3^ Department of Research and Innovation (DAIRI), Azienda Ospedaliero-Universitaria SS. Antonio e Biagio e Cesare Arrigo, Alessandria, Italy

^4^ Department of Health Science, University of Eastern Piedmont, Alessandria, Italy

^5^ Department of HBP and Digestive Oncological Surgery, Robert Debré University Hospital, University Reims Champagne-Ardenne, France

^6^ Institut de Recherche sur les Maladies Virales et Hépatiques, U1110, Université de Strasbourg, Strasbourg, France

**Corresponding Author**

Fabio Giannone, MD, PhD

Robotic, Oncologic and HPB Surgery

University Hospital of Alessandria

Via Venezia 16

15121 Alessandria, Italy

Phone number : +39 0131 207485

Email: [fabio.giannone@ospedale.al.it](mailto:fabio.giannone@ospedale.al.it)

**SUPPLEMENTARY TABLE 1**

| **Supplementary Table 1.** Clinico-pathological features according to *KRAS* mutational status | | | | | |  |
| --- | --- | --- | --- | --- | --- | --- |
| **Variables** | **Whole cohort,**  **n= 220** | ***KRAS* wild-type,**  **n= 146** | ***KRAS* mutated**  **n= 74** | **P** |  |  |
|  | ***n (%)*** | | |  | | |
| Median Age, yrs (IQR) | 66 (57-72) | 65 (22.7-28) | 67 (59-71.2) | 0.095 |  |  |
| Sex  Male  Female | 146 (66.4)  74 (33.6) | 98 (67.1)  48 (32.9) | 48 (64.9)  26 (35.1) | 0.738 |  |  |
| Median BMI, kg/m^2^ (IQR) | 25 (22.7-28) | 25.3 (22.7-28) | 24.8 (22.7-28) | 0.664 |  |  |
| ASA  I  II  III | 13 (5.9)  109 (49.5)  98 (44.5) | 9 (6.2)  69 (47.3)  68 (46.6) | 4 (5.4)  40 (54.1)  30 (40.5) | 0.635 |  |  |
| Status at diagnosis  Synchronous  Metachronous | 140 (63.6)  80 (36.4) | 92 (63)  54 (37) | 48 (64.9)  26 (35.1) | 0.787 |  |  |
| Site of primary tumor  Right colon  Transverse colon  Left colon  Rectum | 57 (25.9)  9 (4.1)  88 (40)  66 (30) | 29 (19.9)  7 (4.8)  63 (43.2)  47 (32.2) | 28 (37.8)  2 (2.7)  25 (33.8)  19 (25.7) | ***0.038*** |  |  |
| Previous primary tumor resection  No  Yes | 41 (18.6)  179 (81.4) | 29 (19.9)  117 (80.1) | 12 (16.2)  62 (83.8) | 0.512 |  |  |
| Preoperative Chemotherapy  No  Yes | 34 (15.5)  186 (84.5) | 27 (18.5)  119 (81.5) | 7 (9.5)  67 (90.5) | 0.080 |  |  |
| CEA (median), ng/ml (IQR) | 107 (2-19) | 3.3 (1.9-10.5) | 8 (3-66.1) | ***0.004*** |  |  |
| Number of nodules  <4  ≥4 | 115 (52.3)  105 (47.7) | 75 (51.4)  71 (48.6) | 40 (54.1)  34 (45.9) | 0.706 |  |  |
| Distribution  Unilobar  Bilobar | 99 (45)  121 (55) | 69 (47.3)  77 (52.7) | 30 (40.5)  44 (59.5) | 0.344 |  |  |
| Largest nodule (mm), median (IQR) | 30 (20-48) | 20 (20-50) | 30 (21-45) | 0.983 |  |  |
| Type hepatectomy  Minor  Major | 130 (59.1)  90 (40.9) | 84 (57.5)  62 (42.5) | 46 (62.2)  28 (37.8) | 0.509 |  |  |
| Approach  Open  MI | 162 (73.6)  58 (26.4) | 104 (71.2)  42 (28.8) | 58 (78.4)  16 (21.6) | 0.256 |  |  |
| TA performed  No  Yes | 105 (47.7)  115 (52.3) | 73 (50)  73 (50) | 32 (43.2)  42 (56.8) | 0.343 |  |  |
| R status  R0  R1 | 189 (85.9)  31 (14.1) | 126 (86.3)  20 (13.7) | 63 (85.1)  11 (14.9) | 0.814 |  |  |
| Primary T status  Complete response  1  2  3  4 | 6 (2.7)  7 (3.2)  23 (10.5)  139 (63.2)  45 (20.5) | 4 (2.7)  7 (4.8)  16 (11)  92 (63)  27 (18.5) | 2 (2.7)  -  7 (9.5)  47 (63.5)  18 (24.3) | 0.346 |  |  |
| Primary N status  0  1  2 | 79 (35.9)  81 (36.8)  60 (27.3) | 56 (38.4)  51 (34.9)  39 (26.7) | 23 (31.1)  30 (40.5)  21 (28.4) | 0.551 |  |  |

LR: Liver Resection; TA: Thermal Ablation; KRAS: Kirsten Rat Sarcoma Virus; IQR: Interquartile Range; BMI: Body-Mass Index; ASA: American Society of Anesthesiologists; CEA: Carcinoembryonic Antigen; MI: Minimally-invasive.

**SUPPLEMENTARY FIGURE**

**Supplementary Figure 1.** Flow-chart of patients’ screening process

**
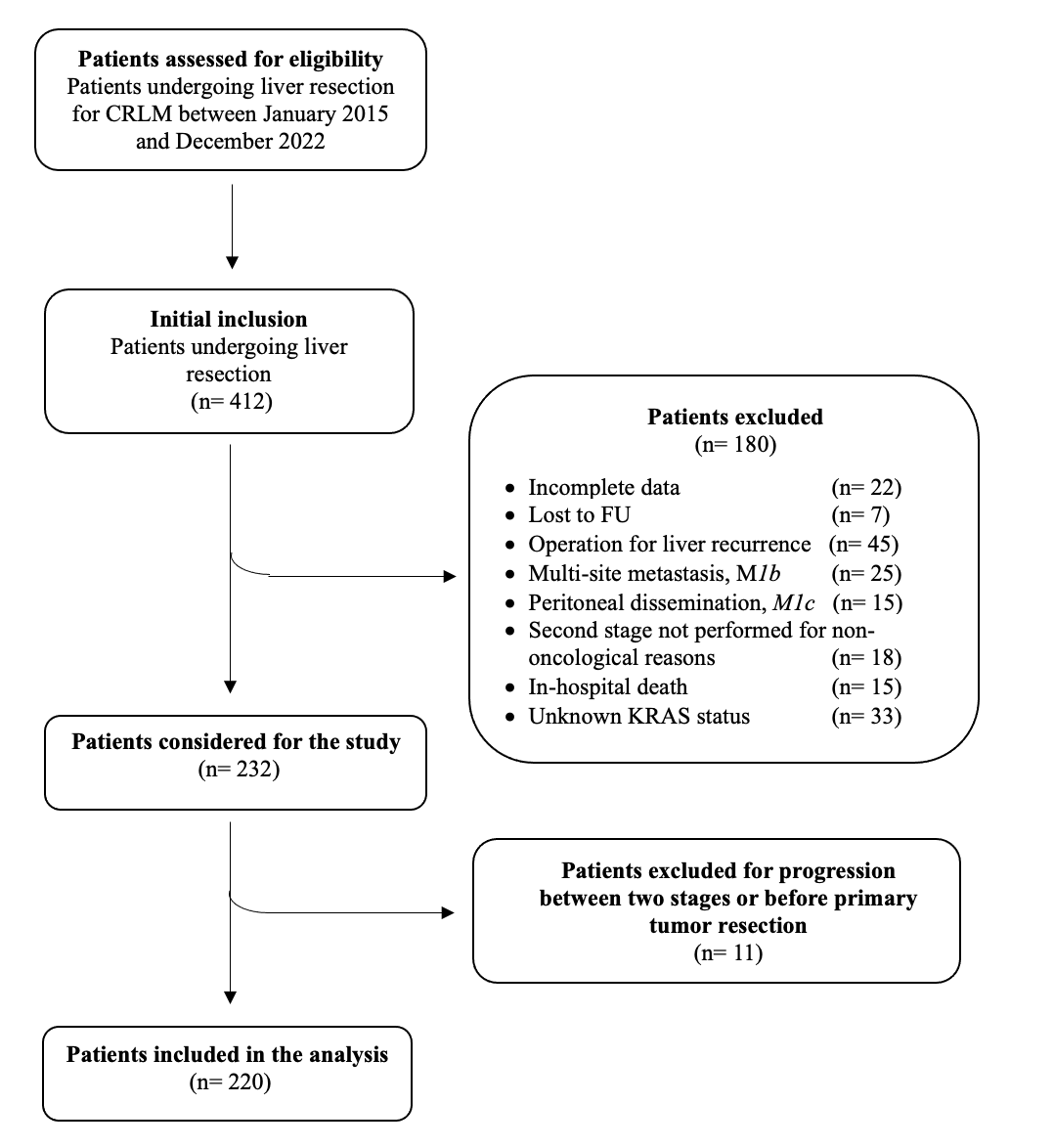
**

**Supplementary Figure 2.** Kaplan-Meier curves of DFS (A) and DSS (B) comparing the two groups (liver resection alone vs liver resection + thermal ablation) in *wtKRAS* patients. LR: Liver Resection, TA: Thermal Ablation.

**
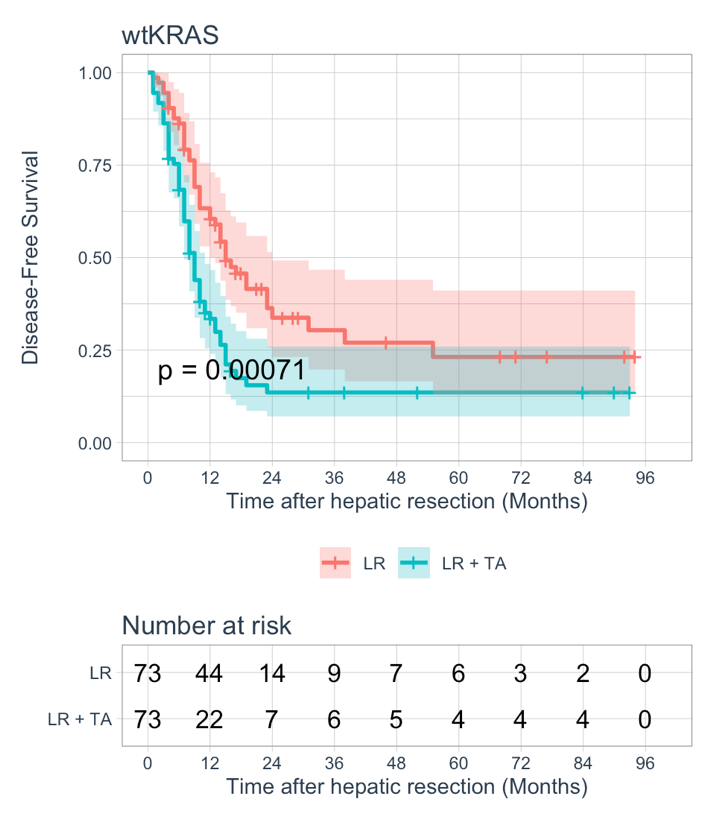

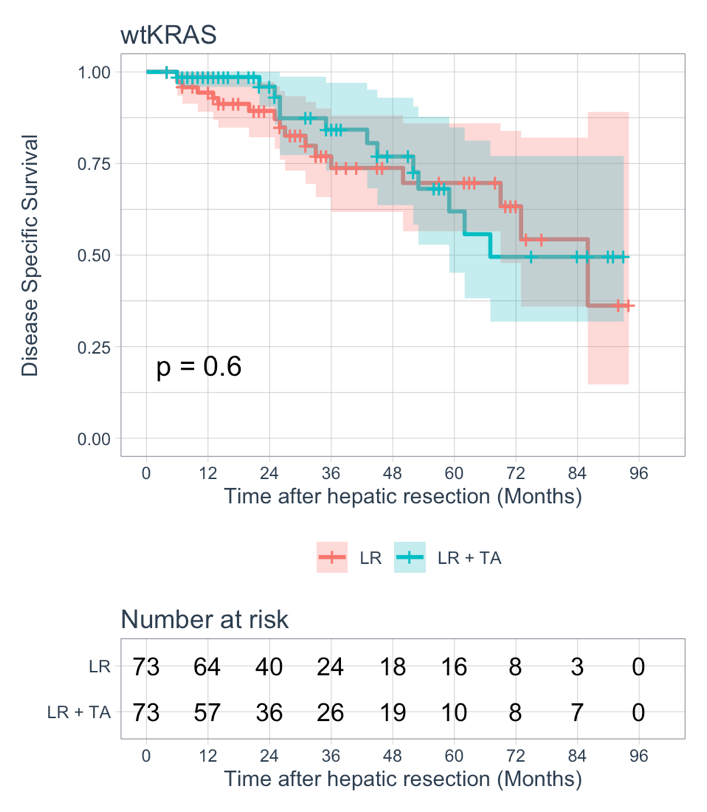
**

**B**

**A**
